# Atomic Force Microscopy Analysis of DNA Extracted from the Vegetative Cells and the Viable, but Nonculturable, Cells of Two Mycoplasmas (*Acholeplasma laidlawii* PG8 and *Mycoplasma hominis* PG37)

**DOI:** 10.1100/tsw.2010.78

**Published:** 2010-05-18

**Authors:** Maxim V. Trushin, Vladislav M. Chernov, Oleg V. Gorshkov, Natalija B. Baranova, Olga A. Chernova

**Affiliations:** ^1^ Kazan Institute of Biochemistry and Biophysics, Russian Academy of Sciences, Kazan, Russia; ^2^ Department of Genetics, Kazan State University, Kazan, Russia

**Keywords:** DNA, mycoplasma, Acholeplasma laidlawii PG8, Mycoplasma hominis PG37

## Abstract

This article reports on a study of some characteristics of DNA extracted from the vegetative and viable, but nonculturable (VBNC), cells of two mycoplasma species (*Acholeplasma laidlawii* PG8 and *Mycoplasma hominis* PG37) using atomic force microscopy (AFM). DNA images were obtained by operating the AFM microscope in the tapping mode. It was found that DNA from the VBNC forms of *M. hominis* PG37 has decreased sizes (height: 0.177 ± 0.026 nm vs. 0.391 ± 0.041 nm for the vegetative forms, and width: 1.92 ± 0.099 vs. 2.17 ± 0.156 nm for the vegetative forms) in comparison to DNA from the vegetative forms of the mycoplasma. In the case of DNA from the *A. laidlawii* PG8 VBNC forms, we detected a decrease in width (1.506 ± 0.076 nm vs. 1.898 ± 0.117 nm for the vegetative forms), but an increase in height (0.641 ± 0.068 nm vs. 0.255 ± 0.010 nm for the vegetative forms) of the molecule. Analyzing the obtained results, one can speculate on some similarities in the physical-chemical properties of DNA from *M. hominis* PG37 and *M. gallisepticum* S6. In turn, this implies some general mechanisms of adaptation to a severe environment.

## INTRODUCTION

The inability of bacteria to grow on various bacteriological media, but saving their own metabolic activity at the same time, is referred to as the viable, but nonculturable (VBNC) state[1]. The VBNC state may be caused by some stressors, including factors of a chemical[2,3] and physical nature[4,5], or their combinations[6,7,8]. Resuscitation of VBNC cells is possible after cessation of these factors[9,10], however, in some cases, VBNC cells cannot be resuscitated[11]. Also, this process may be strain dependent[12] or may rely on environmental microcosms[13,14].

In the VBNC state, bacteria experience several morphological and physiological changes, including cell dwarfing (in particular, a shift from rod to coccoid form with decreased size)[15], alterations in protein expression[16,17] and membrane structure[18], as well as some other modifications[19]. Previously, the analogous phenomena were detected in mycoplasmas (class Mollicutes). While in the VBNC state, mycoplasmas have altered cell shape[20], gene expression[21], pathogenicity[22], and genotoxic features[23].

Different experimental techniques were applied to investigate cells in the VBNC state, including flow cytometry[24,25], solid phase cytometry[26], laser-scanning cytometry[27], as well as scanning and transmission electron microscopy[28]. Together with the above-mentioned methods, atomic force microscopy (AFM) may provide much information on the structure of a whole cell and its components[29,30]. In our previous work devoted to the AFM investigation of the DNA of *Mycoplasma gallisepticum* S6, we detected metric differences in DNA isolated from the vegetative and VBNC forms of the mycoplasma[31]. The aim of this article, as an extension of the previous experiments, was to report on a study of some characteristics of DNA extracted from the vegetative and VBNC cells of two mycoplasma species (*Acholeplasma laidlawii* PG8 and *M. hominis* PG37) using AFM.

## MATERIALS AND METHODS

*Acholeplasma laidlawii* PG8 and *M. hominis* PG37 were obtained from the N.F. Gamalei Research Institute of Epidemiology and Microbiology (Moscow, Russia). *A. laidlawii* PG8 cells were cultivated for 2 days at 37°C on Edward's medium (tryptose, 2% [w/v]; NaCl, 0.5% [w/v]; KCl, 0.13% [w/v]; Tris base, 0.3% [w/v]; serum of horse blood, 10% [w/v]; fresh yeast extract, 5% [w/v]; glucose solution, 1% [w/v]; benzylpenicillin [500,000 IE/ml], 0.2% [w/v]; phenol red, 0.3% [w/v]) to obtain the vegetative cells. Cultivation of *M. hominis* PG37 for 3–5 days at 37°C was performed on the modified Edward's medium containing tryptose, 1.5% (w/v); HEPES, 0.2% (w/v); serum of horse blood, 10% (w/v); fresh yeast extract, 5% (w/v); arginine, 4% (w/v); benzylpenicillin (500,000 IE/ml), 0.2% (w/v); and phenol red, 0.3% (w/v). To obtain VBNC cells, glucose/arginine, serum of horse blood, yeast extracts, and phenol red were eliminated from Edward's medium; microorganisms were kept in this condition up to 13 months.

To extract DNA from *A. laidlawii* PG8 and *M. hominis* PG37 cells, the following procedures were applied. Mycoplasma cultures (the vegetative and VBNC cells) were centrifuged at 9000*g* for 20 min. The pellet was resuspended in TES buffer (Tris-HCl 10 mM, EDTA 1 mM, NaCl 100 mM), SDS (1–2%) was added to the solution, then shaken and stored at 37°C for 5–10 min. An equal volume of aqueous phenol-chloroform mixture (1:1) was added to the obtained cell lysate. The aqueous phase was separated by centrifugation and the second extraction with chloroform-isoamyl alcohol mixture (24:1) was performed. DNA was precipitated with 2.5 volume of ethanol (96%). The pellet was dissolved in TE buffer (Tris-HCl 10 mM, Na-EDTA 1 mM, pH 8.0). For additional purification, the solution was incubated with RNAse (20 μg/mL, Serva, Germany) for 30 min at 37°C and then with proteinase K (50 μg/mL, Sigma, USA) for 30 min at 37°C. After the enzymatic treatment, DNA was again extracted with chloroform-isoamyl alcohol mixture and precipitated with 2.5 volume of ethanol (96%). DNA concentration was detected in agarose gel electrophoresis using standard DNA samples (Fermentas, Lithuania). The applied method allowed us to obtain DNA fragments that were then analyzed using AFM. A buffer containing 5 mM $MgCl_2$ and 10 mM Tris was used to dilute the obtained DNA samples to specific concentrations (0.15–0.5 ng/μL). Since mica has a multilayer structure, it is necessary to eliminate the upper layer before coating samples. The upper layer of mica (Advanced Technologies Center, Moscow, Russia) was eliminated with tape. For this purpose, 1- × 1-cm mica squares were placed onto the bottom of a 3-cm plastic Petri dish and covered superiorly with tape. When the tape was taken off, the upper layer of mica was eliminated. DNA samples (3 μL) were placed onto the mica for 1 min.

Then the mica with DNA was rinsed twice with redistilled water and after each rinsing, it was dried with pressurized air. Images were acquired in air by a Solver P47H atomic force microscope (NT-MDT, Moscow, Russia) operated in the tapping mode using fpN11S cantilevers ($r \leq 10$ nm, Advanced Technologies Center, Moscow, Russia). The height, Mag (signal from lock-in amplifier), RMS (signal from RMS detector), and phase (signal from the phase detector) were performed with the Nova 1.0.26 RC1 software (NT-MDT, Moscow, Russia). DNA height was measured directly using height regime in Nova 1.0.26 RC1. DNA width at the half-maximum was seen on the section of the DNA molecule with the compilation adjustment performed with the Nova 1.0.26 RC1 software.

The Shapiro-Wilk test was applied to test whether our data yielded a normal distribution. Significant differences in the results were evaluated by applying Student's *t* test. A *p* value of <0.05 was considered significant. Data are given as mean ± SE. All calculations were made using the Origin 8.0 software for Windows.

## RESULTS AND DISCUSSION

AFM is widely used to investigate DNA and DNA-protein complexes[32,33]. However, mycoplasma DNA was not studied with AFM apart from one exception[31]. In this article, we provide new data on DNA molecules isolated from vegetative and VBNC cells of *A. laidlawii* PG8 and *M. hominis* PG37.

Fig. 1 presents the noncontact, tapping mode, AFM images of DNA taken from *M. hominis* PG37 and *A. laidlawii* PG8 cells grown in different physiological states. For the analysis, DNA regions of the molecules (approximately 200-nm long absorbed on the specially prepared mica surfaces) were chosen. We report here for the first time that the above-mentioned DNA parameters differ in the molecules when they are extracted from vegetative vs. VBNC cells. Fig. 1 clearly demonstrates differences in DNA height in the used scanning regime. It should be kept in mind that the intensity of coloring (in our case, pompadour color above the navy blue background) reflects the parameter of height. There is a positive correlation (intensive coloring corresponds to increased values of the height parameter). One can see that the most intensive coloring was seen for DNA molecules extracted from VBNC forms of *A. laidlawii* PG8 (Fig. 1D), while the least intensive coloring was detected for DNA from VBNC forms of *M. hominis* PG37. This fits with the numerical data presented in Table 1. To compare the obtained data statistically, we used Student's *t* test. However, before using this statistical criterion, we found that all sets of experimental data fit to normal distribution (see explanations for Table 1). Therefore, the application of Student's *t* test was proper. It is clear from Table 1 that DNA height and width have reduced values when the molecules were extracted from VBNC forms of *M. hominis* PG37 cells. Namely, there was an approximately twofold reduction in height ($t = 12.788$, $p < 0.01$) and a slight (but statistically significant) reduction in width ($t = 3.217$, $p < 0.05$). However, we detected a slight (but statistically significant) reduction in DNA width ($t = 3.194$, $p < 0.05$) in VBNC forms of *A. laidlawii* PG8, while DNA height was increased ($t = 7.958$, $p < 0.01$). In schematic form, the detected changes in DNA height and width are presented in Fig. 2.

It is therefore reasonable to suggest that changes in cell physiology are consequences of alterations in the topology features of DNA. We previously reported that DNA of another mycoplasma, *M. gallisepticum* S6, had altered height and width values when the molecule was extracted from VBNC forms[31]. The latter finding corresponds with our current data for DNA from *M. hominis* PG37, but does not for *A. laidlawii* PG8. In cases of *M. hominis* PG37 and *M. gallisepticum* S6, reductions in DNA height and width were detected when the DNA molecule was isolated from VBNC forms, but in the case of *A. laidlawii* PG8, increasing height and decreasing width of the DNA molecule was registered. DNA determines the level of gene expression by virtue of its interaction with regulatory proteins[34,35]. It should be noted here that trends on modulation of protein expression in *M. gallisepticum* S6, owing to comparative proteomic analysis of the vegetative and VBNC forms, proved to be quite similar for *M. hominis* PG37, but not for *A. laidlawii* PG8 (unpublished data).

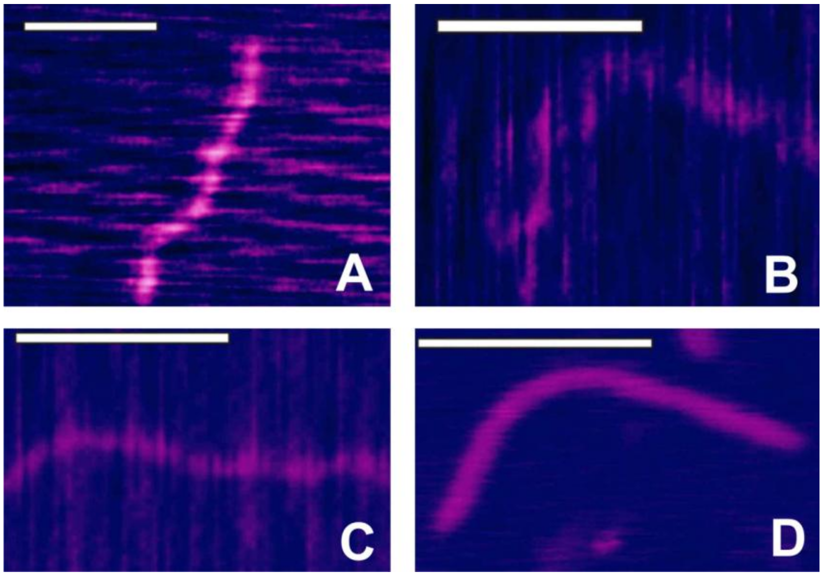

**FIGURE 1**. Noncontact, tapping mode, AFM images of DNA obtained from *M. hominis* PG37 (A, B) and *A. laidlawii* PG8 (C, D) cells in air. Note: the vegetative forms (A, C) and VBNC forms (B, D) are shown. The presented images were recorded in height regime. Bars correspond to 100 nm.

**TABLE 1**
**The Measured Height and Width of the DNA Molecules Extracted from *A. laidlawii* PG8 and *M. hominis* PG37**

| DNA Parameter (nm) | Vegetative Form | | VBNC Form | |
|---|---|---|---|---|
| | ***A. laidlawii* PG8** | ***M. hominis* PG37** | ***A. laidlawii* PG8** | ***M. hominis* PG37** |
| Height | 0.255 ± 0.01 (0.869) | 0.391 ± 0.041 (0.991) | 0.641 ± 0.068 (0.645) | 0.177 ± 0.026 (0.978) |
| Width | 1.898 ± 0.117 (0.902) | 2.17 ± 0.156 (0.808) | 1.506 ± 0.076 (0.93) | 1.92 ± 0.099 (0.718) |

*Note*: Data are given as mean ± standard error of the mean. DNA parameters (height and width of the molecule) are presented in nm. The measurements of DNA parameters at the chosen regions were repeated 20 times (n = 20). After each DNA value, meanings of Shapiro-Wilk test are indicated in brackets. See clarifications in the text.

About 20 years ago, the paranemic model for DNA was suggested by Dr. Clive Delmonte[36]. This model implies an untwisted DNA structure contrary to a twisted structure in the Watson-Crick model. According to paranemic model by Delmonte, the sugar-phosphate backbones are antiparallel, right-handed helices, but they do not cross one another. Therefore, the two strands are topologically nonintertwined[37,38]. Contrary to the Watson-Crick model, the angular relationships between the bases and the sugar-phosphate backbone are not the same. In the model proposed by Delmonte, each of the 10 bases in the repeat unit of the structure has a slightly different angular relationship with the sugar-phosphate backbone of the DNA molecule. The last feature of the DNA molecule may account for the observed DNA changes. For example, DNA heights of around 0.4 to 0.7 nm represent stacks of base pairs edge on to the surface, and the smaller heights, around 0.17 to 0.18 nm, could be base pairs, which have been pushed over. Thus, if that is accepted, changes in DNA topology (probably at the level of the nucleotide locations within

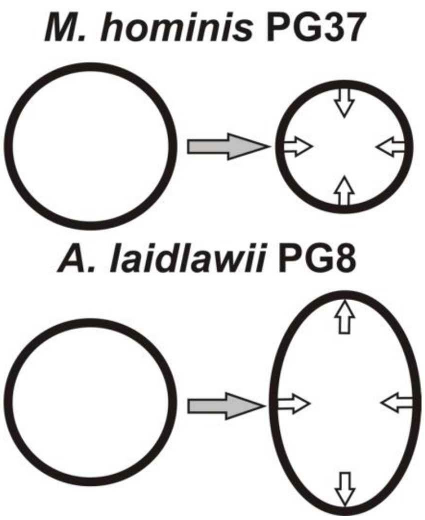

**FIGURE 2.** Scheme of the detected changes in DNA molecules of *M. hominis* PG37 and *A. laidlawii* PG8. *Note*: circles at the left side of the image are schematic illustrations of cross-sections of DNA molecules from the indicated bacteria grown in normal conditions (the vegetative forms). Arrows indicate changes observable in DNA isolated from VBNC forms of the corresponding mycoplasma cells. In *M. hominis* PG37, we detected "compression" of DNA: reductions in height and width of the molecule (the small circle at the upper right part of the image). In *A. laidlawii* PG8, we detected reduction in width, but increasing height of DNA (oval at the lower right part of the image).

the antiparallel single helices) are primary happenings in the chain of adaptation events. This may imply that similar DNA alterations for *M. hominis* PG37 and *M. gallisepticum* S6 may be responsible for the corresponding mechanisms of adjustment of the mycoplasmas to unfavorable growth conditions. Probably, *A. laidlawii* PG8 has a distinct mechanism of adaptation that reflects at the level of DNA by the different "molecule behavior". This suggestion should be tested in further experiments.

## ACKNOWLEDGMENTS

This work was supported by the grant of the President of the Russian Federation (MK-3372.2009.4), grant of the Russian Fund for Basic Research (Project #08-04-01047a), and Government contract # 02.740.11.0391. We thank Dr. Olga A. Konovalova and Mr. Dmitry Nalimov (Kazan State University, Department of Physics) for their help during work on the atomic force microscope. Also, we are very grateful to Dr. Clive S. Delmonte (Open University, Clarendon Park, Nottingham, U.K., Retired) for useful discussions.
